# Longitudinal Study of Fractional Exhaled Nitric Oxide (FeNO) and Spirometric Indices in Trainee Bakers and Confectioners

**DOI:** 10.1111/cea.70076

**Published:** 2025-05-14

**Authors:** Gina‐Maria Klaut, Ludwig Frei‐Stuber, Stefan Karrasch, Susanne Kutzora, Jonas Huss, Doris Gerstner, Dennis Nowak, Caroline Quartucci

**Affiliations:** ^1^ Institute and Clinic for Occupational, Social and Environmental Medicine University Hospital, Ludwig‐Maximilians‐Universität (LMU) Munich, Comprehensive Allergy Center Munich (CAC‐M) Munich Germany; ^2^ Comprehensive Pneumology Center Munich (CPC‐M) Member of the German Center for Lung Research (DZL) Munich Germany; ^3^ Institute of Epidemiology Helmholtz Zentrum München—German Research Center for Environmental Health Neuherberg Germany; ^4^ Bavarian Health and Food Safety Authority Institute for Occupational Health and Product Safety, Environmental Health Munich Germany

**Keywords:** allergy, baking industry, bronchial asthma, early detection, flour dust, fractional exhaled nitric oxide, occupational diseases, prevention, rhinitis, spirometry

**Summary**

- Longer duration of exposure to flour dust was associated with higher FeNO levels.
- Spirometric indices decreased with longer duration of flour dust exposure during one school year.

To the Editor,

Allergic diseases (e.g., bronchial asthma and rhinitis) remain common occupational diseases involving exposure to flour allergens [1]. Allergic symptoms mostly occur during the apprenticeship for bakery [2].

Multiple factors influencing fractional exhaled nitric oxide (FeNO) results have been described [3, 4]. Optimal FeNO cut‐off values for the diagnosis of asthma in adults range from 15 ppb to 64 ppb (sensitivity 29%–79%, specificity 55%–95%) [4].

Our aim was to examine associations between the exposure to baking ingredients, FeNO, and spirometry, taking into account nonoccupational influences.

We performed a longitudinal study (observation period from September 2021 to July 2022) carried out at a Vocational School of Bakery and Confectionery in Germany. During each visit (intervals of preferably 3–5 weeks), subjects' health status and workplace conditions were assessed by questionnaires and spirometry (using Global Lung Initiative reference values (2012)) [5], and FeNO measurements were performed. The main aspects of the questionnaires are listed in Table 1. Details on the study design have been described earlier (Klaut, G.‐M. et al. (2024)) [6].

**TABLE 1:** Characteristics of the study population stratified for the two types of apprenticeship bakery and confectionery.

Participants (*n*)	Trainee bakers	Trainee confectioners	Total
	50	77	127
Gender (*n*, %)			
Female	18 (36.0)	64 (83.1)	82 (64.6)
Male	32 (64.0)	13 (16.9)	45 (35.4)
Age (median, interquartile range 25%‐75%)^a^	20.5 (18.0‐24.0)	20.5 (18.0‐22.0)	20.0 (18.0‐23.0)
Year of apprenticeship (*n*, %)			
First year	16 (32.0)	30 (39.0)	46 (36.2)
Second year	15 (30.0)	21 (27.3)	36 (28.3)
Third year	12 (24.0)	17 (22.1)	29 (22.8)
Missings	7 (14.0)	9 (11.7)	16 (12.6)
Allergy diagnosed by a physician (*n*, %)			
Yes	5 (10.0)	17 (22.1)	22 (17.3)
No	45 (90.0)	60 (77.9)	105 (82.7)
At least one respiratory infection (*n*, %)^b^			
Yes	15 (30.0)	34 (44.2)	49 (38.6)
No	35 (70.0)	43 (55.8)	78 (61.4)
Anti‐inflammatory medication (*n*, %)^b^			
Antihistamines	1 (2.0)	2 (2.6)	3 (2.4)
NSAIDs	2 (4.0)	—	2 (1.6)
Steroids	—	—	—
None	47 (93.0)	75 (97.4)	122 (96.1)
Smoking (*n*, %)^b^			
Yes	17 (34.0)	18 (23.4)	35 (27.6)
No	33 (66.0)	59 (76.6)	92 (72.4)

^a^ At the beginning of the study.

^b^ During study.

For the consideration of both occupational and non‐occupational influences on FeNO as well as the *z*‐scores of forced expiratory vital capacity (FVC), forced expiratory volume in 1 s (FEV1) and FEV1/FVC ratio, Generalized Linear Mixed Models (GLMM) analyses for longitudinal analysis were implemented. All study participants were included in the GLMM analyses. The calculation models took the duration of exposure, profession (bakery/confectionery), respiratory infection, allergy, tobacco consumption, vapor extraction systems, low‐dust flour and transfer of flour with low dust development into account. The outcome FeNO was logarithmically transformed to approximate a normal distribution and the delogarithmized values of the coefficients of the predictors of FeNO were reported as e^β^. A significance level of *α* = 0.05 was chosen in all analyses. SAS 9.4 (SAS Institute Inc., Cary, NC) was used to fit GLMM.

Descriptive statistics are presented in Table 1.

Very rarely, relevant exposures to flour dust were reported during the first examination before the start of apprenticeship (baker apprentices: *n* = 2; confectionery apprentices: *n* = 1). No additional non‐occupational exposure to flour dust was reported.

The number of participants decreased during the course of the study. Regarding the trainee bakers, in the beginning, 50 took part in the measurement of FeNO and 33 performed spirometry; after 24–26 weeks, there were only four participating in the determination of FeNO and three carrying out spirometry. The situation among the confectionery apprentices was similar. Overall, no clinically relevant changes were found.

GLMM analysis revealed a significant increase of FeNO with more days of exposure to flour dust. Due to the utilisation of a logarithmic transformation, the e^β^‐value of 1.0005 indicates an approximate daily increase in FeNO of 0.05%, which corresponds to an annual increase of approximately 19.3%. In the analysis of the influence of respiratory infections, allergy, and tobacco consumption on FeNO, only diagnosed allergy showed a significant association. The use of low‐dust flour resulted in a significantly lower FeNO.

All *z*‐scores of spirometric parameters significantly decreased with longer exposure adjusted for respiratory infection, allergy, smoking, and safety measures. Higher *z*‐scores of FEV1 and FVC, respectively, were associated with installed vapor extraction systems. Moreover, there was a significant relation between a decline of the *z*‐scores of FVC and the use of low‐dust flour.

Overall, the present study showed a significant increase of FeNO and a significant decrease of the *z*‐scores of FVC, FEV1, and FEV1/FVC ratio with increasing duration of exposure to flour dust. This indicates the need of implementing improved occupational safety conditions and occupational health checks during apprenticeship. It is therefore recommended to carry out spirometry and FeNO determination initially (baseline) and regularly (first follow‐up after at least one year). Furthermore, the association of vapor extraction systems with higher *z*‐scores of both FVC and FEV1 in our analysis indicates a preventive benefit of this measure.

Previous studies found no relationship between FeNO levels and the time working in a bakery [7, 8]. However, the examined bakers usually had significantly longer exposure times (Olivieri et al.: Mean age 40.2 years, only bakers, observation period 11 years; Crivellaro et al.: Mean age 39.9 years, only bakers, mean exposure time 14.1 years).

Our observation of lower FeNO in settings with low‐dust flour use indicates the preventive effectiveness of this established measure [9].

Our results showed a decline of the *z*‐scores of FVC, FEV1, and FEV1/FVC ratio in association with increasing time since first exposure to flour dust.

An examination by Crivellaro et al. reported a lower FEV1/FVC ratio with longer exposure time (overall *p* = 0.02) [8]. While direct comparability is limited due to differences in study design and study population, the decrease of FEV1/FVC ratio was in line with our results.

Surprisingly, however, the use of low‐dust flour was associated with lower FVC *z*‐scores and, by trend, also with lower FEV1 *z*‐scores (*p* = 0.0667). A possible explanation could be the simultaneous use of other occupational safety measures not being represented in this calculation (e.g., wearing of respiratory masks).

Based on our results, we recommend regular occupational health check‐ups including spirometry and FeNO measurements for early detection of occupational allergic diseases during apprenticeship. Optimised occupational safety measures (e.g., installation of vapor extraction systems) are recommended.

## Author Contributions

All authors have significantly contributed to the work as presented. Gina‐Maria Klaut: Collection of data by using questionnaires, FeNO, and spirometry measurements. Ludwig Frei‐Stuber: Registration of the study, analysis of descriptive statistics, and writing of the manuscript. Stefan Karrasch: Professional advice throughout the entire study. Susanne Kutzora: Professional advice throughout the entire study. Jonas Huss: Calculation of Generalised Linear Mixed Models and professional advice concerning their interpretation. Doris Gerstner: Professional advice regarding Generalised Linear Mixed Models. Dennis Nowak: Professional advice throughout the entire study and correction of the manuscript. Caroline Quartucci: Professional advice throughout the entire study, obtaining the ethics vote, scientific leadership, and correction of the manuscript.

## Disclosure

Registration of the study: The study was registered at the German Register of Clinical Studies of the Federal Institute for Drugs and Medical Devices, Bonn and Cologne (code: DRKS00036088) as well as at the Central Study Register of the Ludwig‐Maximilians‐Universität Clinic, Munich (code: 103114).

## Ethics Statement

The study was approved by the Ethics Committee at the Medical Faculty of Ludwig‐Maximilians‐Universität (approval code: 21–0289), Munich, Germany on May 4th, 2021, and was performed in accordance with the ethical standards of the 1964 Helsinki Declaration.

## Consent

All participants included in the study provided written informed consent. If the subjects were younger than 18 years, the written consent of their legal guardian was additionally required before they could become participants.

## Conflicts of Interest

The authors declare no conflicts of interest.

## Data Availability

The data that support the findings of this study are available upon reasonable request as far as legally possible.
